# Nanotechnology-based approach for safer enrichment of semen with best spermatozoa

**DOI:** 10.1186/s40104-018-0307-4

**Published:** 2019-02-09

**Authors:** Casey L. Durfey, Sabrina E. Swistek, Shengfa F. Liao, Mark A. Crenshaw, Henry J. Clemente, Rooban V. K. G. Thirumalai, Christy S. Steadman, Peter L. Ryan, Scott T. Willard, Jean M. Feugang

**Affiliations:** 1 Department of Animal and Dairy Sciences, Mississippi State University, Mississippi State, MS USA; 2 Department of Biochemistry, Molecular Biology, Entomology and Plant Pathology, Mississippi State, MS USA; 3 Clemente Associates, Madison, CT USA; 4 Institute of Imaging and Analytic Technology (I2AT), Mississippi State University, Mississippi State, MS USA; 5 Department of Pathobiology and Population Medicine Biochemistry, Mississippi State University, Mississippi State, MS USA

**Keywords:** Acrosome reaction, Apoptosis, Artificial insemination, Boar, Iron oxide nanoparticles, Nanopurification, Nanoselection, Nanotechnology, Reproduction, Swine

## Abstract

**Background:**

Advances in nanotechnology have permitted molecular-based targeting of cells through safe and biocompatible magnetic nanoparticles (MNP). Their use to detect and remove damaged spermatozoa from semen doses could be of great interest. Here, MNP were synthesized and tested for their ability to target apoptotic (annexin V) and acrosome-reacted (lectin) boar spermatozoa, for high-throughout retrieval in a magnetic field (nanoselection). The potential impacts of nanoselection on sperm functions and performance of offspring sired by sperm subjected to nanoselection were determined. Fresh harvested and extended boar semen was mixed with various amounts (0, 87.5, and 175 μg) of MNP-conjugates (Annexin V-MNP or Lectin-MNP) and incubated (10 to 15 min) for 37 °C in Exp. 1. In Exp. 2, extended semen was mixed with optimal concentrations of MNP-conjugates and incubated (0, 30, 90, or 120 min). In Exp. 3, the synergistic effects of both MNP-conjugates (87.5 μg – 30 min) on spermatozoa was evaluated, followed by sperm fertility assessments through pregnancy of inseminated gilts and performance of neonatal offspring. Sperm motion, viability, and morphology characteristics were evaluated in all experiments.

**Results:**

Transmission electron microscopy, atomic force microscopy, and hyperspectral imaging techniques were used to confirm attachment of MNP-conjugates to damaged spermatozoa. The motility of nanoselected spermatozoa was improved (*P* < 0.05). The viability of boar sperm, as assessed by the abundance of reactive oxygen species and the integrity of the acrosome, plasma membrane, and mitochondrial membrane was not different between nanoselected and control spermatozoa. The fertility of gilts inseminated with control or nanoselected spermatozoa, as well as growth and health of their offspring were not different between (*P* > 0.05).

**Conclusions:**

The findings revealed the benefit of magnetic nanoselection for high-throughput targeting of damaged sperm, for removal and rapid and effortless enrichment of semen doses with highly motile, viable, and fertile spermatozoa. Therefore, magnetic nanoselection for removal of abnormal spermatozoa from semen is a promising tool for improving fertility of males, particularly during periods, such as heat stress during the summer months.

## Background

Numerous factors associated with the boar (e.g., genetic, health, nutrition, etc.), the environment (e.g., seasonal variations, stress, etc.), and the post-collection semen manipulation (e.g., cryopreservation, etc.) are known to affect sperm quality and fertility potential [1, 2]. Consequently, the causes of poor semen quality are multifactorial [3, 4], and reactive oxygen species (ROS), excreted by non-viable spermatozoa within the semen, are harmful to intact spermatozoa [5, 6]. The removal of non-viable or damaged spermatozoa from semen doses is essential to maintain high reproductive performance of males. To this end, the current progress in nanotechnology gives new prospects to develop novel non-destructive and non-invasive techniques for sperm manipulation in livestock [7, 8].

Nanotechnology is a new field of science dealing with molecules less than 100 nm in diameter, known as nanoparticles. Their unique physico-chemical properties and tunable synthesis make them suitable tools for various bio-applications [7, 9], with promising potentials in reproductive sciences [10–14]. The use of nanoparticles to target physical and physiological characteristics of sperm (motility, directionaliy, apoptosis, intact acrosome, etc.) can help predict whether a semen sample is suitable for assisted reproductive techniques (ART), leading to successful fertilization [15, 16]. Routine techniques for sperm purification such as swim-up [17], discontinuous percoll [18], albumin filtration [19], density gradient centrifugations (DGC) [8], and magnetic-assisted cell sorting (MACS) [20, 21] yield low numbers of motile spermatozoa, but appear more suitable for small-scale applications such as in vitro fertilization (IVF) and intra-cytoplasmic sperm injection (ICSI). Comparative studies have revealed the preponderant effects of MACS for the selection of viable spermatozoa [16, 20], leading to better reproductive outcomes than other techniques (i.e., DGC). Despite the numerous advantages of MACS (e.g., simple, rapid, affordable), its performance is limited to less than 10^9^ spermatozoa processed for a single target of sperm viability parameter (i.e., apoptosis) [16, 22], which drastically limits its applicability in the swine industry.

Therefore, the use of silane- and polyvinylpyrrolidone-coated colloid silica in density (DLC) and single (SLC) layer centrifugation protocols has revealed beneficial for selecting high quality spermatozoa [23–26], through the purification of high sperm number per analysis (i.e., up to 100 × 10^6^ boar spermatozoa). However, the molecular mechanism of the SLC technique remains unclair [23], and its cost and low recovery yield may limit routine applications in swine farms [24, 27]. Recent studies have reported the use of conjugated magnetic nanoparticles as novel tools for molecular-based selection of spermatozoa regardless of the species [28–30]. These later studies used iron oxide nanoparticle conjugated with annexin V, to target early apoptotic spermatozoa through the externalized phosphatidylserine residues on their surface membrane and lectin, to bind carbohydrates on prematurely acrosome-reacted spermatozoa [22, 29, 31]. Indeed, both apoptosis and acrosome reaction are common defects that characterize non-viable spermatozoa due to increased ROS levels influencing cellular metabolism [32, 33]. The simultaneous targeting of both sperm defects for removal from semen doses would be of great interest in swine farms.

Despite these efforts, swine producers are still unwilling to accept these technologies, and research should continue toward the most acceptable and friendly protocol for substantial reduction of productivity losses in swine farms [3, 34]. Here we used magnetic iron oxide nanoparticles (MNP) that were conjugated to annexin V or lectin to allow a two-step procedure removal of both apoptotic and acrosome-reacted spermatozoa from boar semen doses. Synthesized MNP conjugates were characterized and their effectiveness to interact with boar spermatozoa was confirmed in various exposure conditions (dose-dependent effects and time-dependent incubations). The preservation of sperm function following nanoselection was evaluated both in vitro, through sperm motion and viability characteristics and in vivo, through field fertility trial and neonatal offspring performance.

## Methods

### Reagents, nanoparticle synthesis, semen and animal care

Iron oxide or magnetic nanoparticles (MNP) were synthesized and coated by Clemente Associates (Madison, CT, USA) following an undisclosed proprietary protocol. Coating with lectins (PNA/ LCA) or annexin V (Sigma Aldrich; St Louis, MO, USA) allowed MNP to selectively bind with glycans exposed by damaged acrosomal membranes or early apoptotic spermatozoa, respectively. Stock solutions (1 mg/mL = ~ 2.5 × 10^6^ particles/mg) of each Annexin V-MNP and Lectin-MNP, containing sodium azide were stored at 4 °C until use. Insemination doses (~ 3–4 × 10^9^ spermatozoa/80 mL) of freshly extended boar semen (Yorkshire × Duroc) were purchased (Prestage Farms; West Point, MS, USA) for experiments. Purchased mature crossbred gilts (Yorkshire × Duroc; Prestage Farms) were maintained at the Leveck Animal Research Center (Mississippi Agricultural and Forestry Experiment Station, Mississippi State University) and fed with ad libitum access to water. Animal handling was performed according to protocols approved by the Institutional Animal Care and Use Committee of Mississippi State University. All other reagents were purchased from Sigma-Aldrich.

### Characterization of synthesized MNP conjugates

Aliquots of each synthesized Lectin-MNP and Annexin V-MNP conjugates were placed on transmission electron microscope (TEM) grids and allowed to dry for at least one day. Thereafter, loaded grids were submitted to imaging (TEM-JEOL 2100, 200 kV; instrument with Gatan Orius 832 camera).

### Exp. 1: Dose-dependent targeting of spermatozoa and nanoselection

#### Dose-dependent targeting

Various amounts of each MNP conjugate (0, 87.5, or 175 μg), corresponding to 0, 87.5, or 175 μL of stocks were tested to determine the optimal amount of each MNP conjugate necessary to achieve effective nanoselection. These volumes corresponded to 0, ~ 5500 and ~ 11,000 particles/mL of spermatozoa (40–50 ×10^6^/mL).

#### Nanoselection procedure

**Fig. 1:**
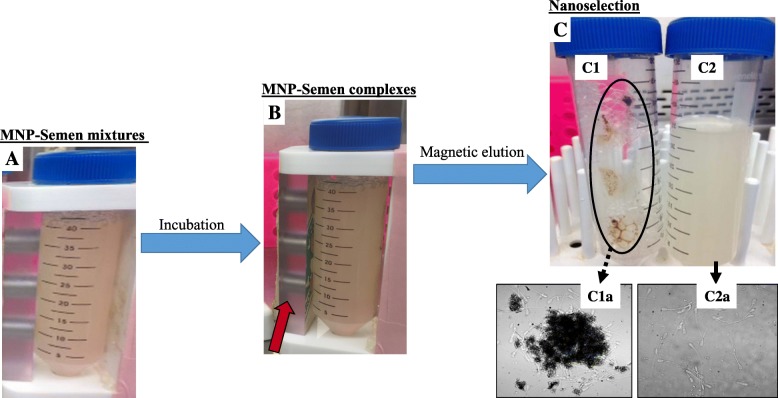
Simplified illustration of the semen nanoselection process. **a** Mixture of extended boar semen with MNP conjugates, then incubation. **b** Sperm-MNP complex formations after interactions between damaged spermatozoa and MNP conjugates, allowing separation under an electromagnetic field (red arrow). **c** Magnetic trapping of sperm-MNP complexes on the tube wall (ellipse) and passive elution of unbound (free) spermatozoa in a new tube (right). Optical microscopy of Sperm-MNP conjugate complexs (C1a) and nanoselected, free MNP conjugate spermatozoa (C2a). For the proposed two-step nanoselection, the described process was performed on same semen samples, starting with Annexin V-MNP conjugates

An illustration of the sperm nanoselection process is shown in Fig. 1. This process consisted to the specific targeting and removal of apoptotic or acrosome-reacted spermatozoa, using Annexin V- or Lectin-conjugated MNP, respectively. For each individual MNP conjugate, freshly harvested and extended boar semen samples (40 mL) were mixed with Annexin V- or Lectin-conjugated MNP (Fig. 1a), incubated for 10–15 min at 37 °C with a gentle rotation, and placed against a strong (12,000 gauss) neodymium magnet, for 10 min at room temperature, to entrap free MNP conjugates and sperm-MNP complexes (Fig. 1b). Unbound or nanoselected (non-apoptotic or acrosome intact) spermatozoa were eluted into new identified tubes (Fig. 1c) for motility and viability analyses. The experiment was repeated at least four times for each MNP conjugate, using single boar semen doses (71.2% ± 1.1% total motility).

### Exp. 2: Incubation time for optimal sperm labeling and nanoselection

Freshly harvested and extended boar semen were incubated with the optimal amount of each MNP conjugate (from Exp.1) and incubated 0, 30, 90, or 120 min at 37 °C. All procedures were performed as described in Exp. 1, followed by motility, viability, and morphology analyses to determine the optimal co-incubation time. The experiment was repeated at least four times for each MNP conjugate, using single boar semen doses (72.0% ± 0.6% total motility).

### Exp. 3: Evaluation of the two-step sperm targeting and nanoselection

#### Two-step targeting for nanoselection

The 1-step procedure corresponded to the removal of apoptotic spermatozoa from extended fresh semen (40 mL) that were mixed with the optimal amount (87.5 μg) of Annexin V-conjugated MNP and incubated for the optimal time (30 min). Thereafter, the mixtures were submitted to nanoselection, as shown in Fig. 1. Resulting nanoselected (non-apoptotic) spermatozoa were eluted into new identified tubes (1-step), and immediately mixed with the optimal amount of Lectin-conjugated MNP for the optimal incubation time to target and remove acrosome reacted spermatozoa. Thereafter, nanoselected spermatozoa (non-apoptotic and acrosome intact) were eluted (2-step) and aliquots were used for various analyses.

#### Maintenance assessment of sperm function

Nanoselected spermatozoa obtained from the two-step procedure, corresponding to 1-step followed by the 2-step were used for various analyses (i.e., motility, viability, and morphology characteristics). In addition, the maintenance of the fertility potential of nanoselected spermatozoa originated from the 2-step procedure was tested.

### Evaluation of sperm-MNP interactions

Aliquots of fresh control or non-selected, MNP-bound (Fig. 1b), and nanoselected (Fig. 1c) spermatozoa were constituted during nanoselection with each MNP conjugate. All samples were subjected to standard preparations for transmission electron (TEM) and atomic/magnetic force (AFM/MFM) microscopes, and hyperspectral imaging (HI) as previously reported [35, 36]. Briefly, samples were fixed 1) and stained with or without lead and uranyl acetate for TEM-JEOL, 2) in 4% paraformaldehyde and smeared on histology microscope slides for AFM/MFM, and 3) on histology glass slides with hyperspectral data collected using reference spectral libraries created by MNP-conjugates (CytoViva® imaging technology; CytoViva Inc., Auburn, AL, USA).

### Analysis of sperm motion and morphology characteristics

Analyses were performed using the HTM-IVOS (Hamilton-Thorne Biosciences; Beverly, MA, USA) or CEROS II (IMV Technologies; Maple grove, MN, USA) Computer-Assisted Sperm Analyzers. Aliquots of nanoselected spermatozoa were submitted to analyses with pre-set values of CASA, as previously described [29]. Each sample aliquot was run in triplicate (3 chambers) with an average of 262 ± 3 spermatozoa analyzed per chamber. Sperm motility [percent of total, progressive, rapid (≥30 μm/s) motility, and static], velocity [μm/s; average path or VAP, straight line or VSL, and curvilinear or VCL], and directionality [lateral head amplitude (ALH, μm), beat cross frequency (BCF, Hz), straightness (STR; VSL/VAP × 100), linearity (LIN; VSL/VCL × 100)] parameters were evaluated. The proportions of morphologically abnormal spermatozoa, such as bent tail (BT), coiled tail (CT), and distal (DD) and proximal (PD) cytoplasmic droplets were also recorded using CEROS II.

### Analysis of sperm viability

Staining protocols adapted from Feugang et al. [37] and Martinez-Alborcia et al. were used [38]. Nanoselected spermatozoa were diluted to 30 × 10^6^ cells/mL with a pre-warmed phosphate-buffered saline solution (PBS). Aliquots of 0.1 mL of sperm suspensions were allocated to various staining for viability assessments: 2 μL propidium iodide (PI, 1 mg/mL in PBS) for plasma membrane integrity, 5 μL PNA-FITC (100 mg/mL in PBS) for acrosomal reaction, 2 μL JC-1 (500 mg/mL; Cayman Chemical Co.; Ann Arbor, MI, USA) for mitochondrial integrity, and 2.5 μL H_2_-DCFDA (1 mmol/L in DMSO) for reactive oxygen species (ROS) accumulation within the cells. All samples were incubated at 37 °C for 15 min, then diluted six times with prewarmed-PBS to reduce both dye and sperm concentrations for adequate and immediate analyses with flow cytometry (Becton–Dickinson FACSDiva version 6.1.3). A total of 10,000 events were set to evaluate the proportions of stained spermatozoa (control and nanoselected) and their relative fluorescence intensities to assess sperm damage. Sample aliquots were mounted onto individual microscope slides to confirm the successful staining under an epifluorescence microscope (EVOS FL-Auto, Thermo Fisher Scientific; Hampton, NH, USA).

### Field fertility test and offspring performance

The fertility maintenance of nanoselected spermatozoa was tested as an indispensable step prior to any large-scale application. Estrus was detected on sexually mature gilts (~ 8 months old) through responsiveness to a teaser boar or a lordosis test (“standing reflex”). Each responding gilt was inseminated twice within 24 h, starting from 6 h post-estrus detection, with single sire doses (two total) of either control (non-selected; *n* = 7 gilts) or nanoselected (*n* = 7 gilts) semen. All inseminated gilts (*n* = 14) were housed in a partially open building during breeding and gestation. Pregnant gilts were later transported to a fully enclosed building for farrowing and lactation within crates. The numbers of pregnant gilts, weaned pigs, and litter sizes were recorded.

Neonatal pigs (1–2 per litter/sperm group) were randomly selected for umbilical blood collection at birth and before nursing. Blood samples of eight neonatal pigs born from each control or nanoselected spermatozoa were harvested into EDTA-coated tubes. Whole blood aliquots were taken for glucose (Glucose meter, Agamatrix Inc., Salem, NH) and pact cell volume or hematocrit analysis, using standard procedures, and the remaining whole blood samples were centrifuged (1500 r/min for 15 min at 4 °C) and blood plasma were collected and stored at -80 °C for immunoglobulin G analyses (porcine IgG ELISA kit; Bethyl Laboratories, Inc.; Montgomery, TX, USA).

The morphometric parameters of all neonate pigs (*n* = 83) were taken weekly, from birth until weaning (~ 28 d post-natal). Weight, growth, crown-rump length (CRL, distance from crown of the head to the base of the tail), body length (distance from tip of snout to base of tail), head length (distance from tip of snout to base of neck), head circumference, and heart girth were measured.

### Statistical analysis

All statistical analysis was performed using the Statistical Analysis Software (SAS) 9.4 (SAS Institute, Inc., Cary, NC). A frequency model was used to determine pregnancy rates, and evaluate fecundity characteristics. A general linear model was used to determine differences between and within control and nanopurified semen in all experiments. A linear mixed model was used to evaluate offspring biochemical analyses from neonatal pigs, with two-way interactions as fixed effects including treatment, and gender. Additionally, a linear mixed model was used to evaluate offspring morphometric analyses from birth until weaning from all neonatal pigs, with treatment, gender, and two-way (treatment × gender and treatment × day) interactions as fixed effects. Repeated measures of morphometric analyses were analyzed using an autoregressive one covariance method. Litter within trial was considered a random effect when applicable. All data are expressed as mean ± standard error mean (SEM), with significant differences set as *P* ≤ 0.05.

## Results

### Characterization of synthesized MNP conjugates

**Fig. 2:**
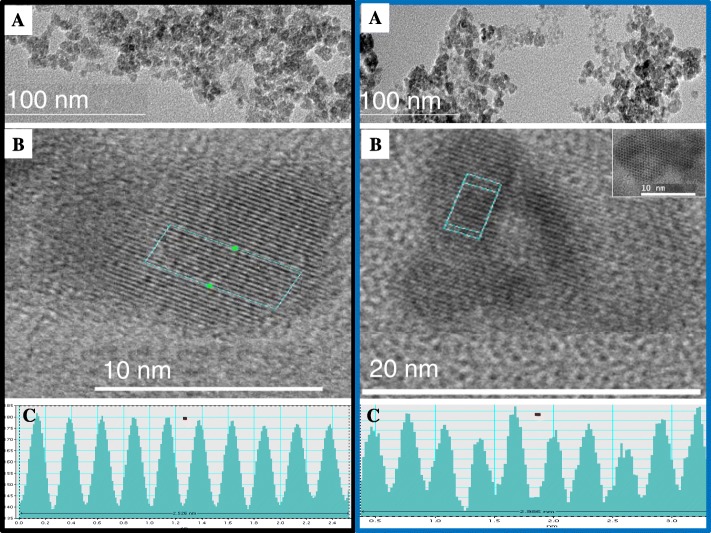
Standard (**A**) and high resolution (**B**) transmission electron microscope of Lectin (Black box)- and Annexin V (Blue box-conjugated MNP. The MNP cores were measured at 7.1 ± 0.2 and 14 ± 0.2 nm (mean ± SEM) in diameters, corresponding to Lectin-MNP and Annexin V-MNP, respectively. The insert in micrograph B shows a lattice interplanar distance or d-spacing of the MNP core. Corresponding lattice line intensity graphs (**C**) revealed average d-spacings of 2.98 Å (hematite or α-Fe_2_O_3_), for Lectin-MNP and 2.53 Å (maghemite or γ-Fe_2_O_3_), for Annexin V-MNP

Results are shown in Fig. 2. The TEM imaging revealed the spherical shape of the synthesized MNP nanoparticles in Fig. 2a, while high resolution (HR) TEM imaging of MNP cores showed planes with interplanar distances (or d-spacing) of 2.53 Å and 2.98 Å (insert in picture B) for Annexin V- and Lectin-MNP, respectively (Fig. 2b and c). Images of both MNP conjugates indicated core diameters of approximately 7.1 ± 0.2 nm and 14 ± 0.4 nm, respectively.

### Evaluation of sperm-MNP interactions

**Fig. 3:**
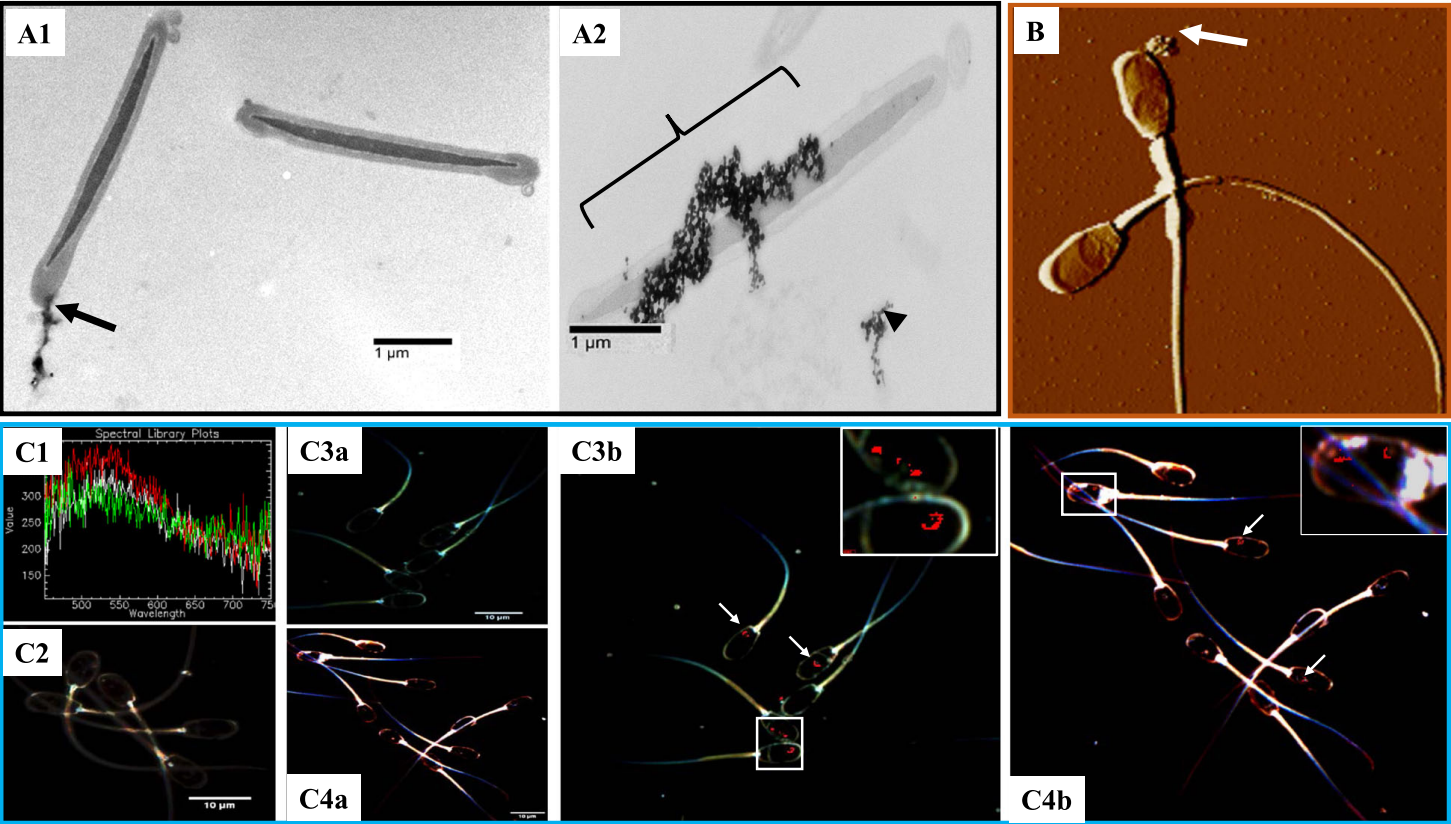
Evaluation of sperm-MNP conjugate interactions, using TEM (**A**), AFM/MFM (**B**), and Hyperspectral (**C**) imaging. Cross-sectioned spermatozoa revealed single-point (A1) and multi-point (A2) attachments of MNP conjugates to spermatozoa. Micrograph indicates Lectin-MNP targeting in the sperm head (arrow) region (B). Hyperspectral library and control boar spermatozoa allowing calibration to detect MNP conjugates are shown in C1 and C2 micrographs, respectively. Micrographs C3a and C4a are optical images of corresponding hyperspectral mapped Annexin V- (C3b) and Lectin- (C3b) MNP appearing as red spots indicated by arrows. Inserts show main localization of MNP within the sperm head

Representative TEM (A), AFM (B), and Hyperspectral (C) imaging are shown in Fig. 3. The TEM imaging revealed single- (A1) and multi-point (A2) attachments of conjugated MNP to sperm membranes. The maintenance of magnetic properties of MNP conjugates interacting with the sperm head, as expected, are seen with AFM/MFM imaging (B). Hyperspectral imaging (C) showed the presence of MNP as red dots, mainly detected within the sperm head region (C3b, for Annexin V-MNP and C4b, for Lectin-MNP). Control (C2) and nanopurified (C3a and C4a) spermatozoa did not show detectable presence of residual MNP.

### Sperm targeting, effectiveness of nanoselection, and sperm motility characteristics

#### Dose-dependent labeling

**Fig. 4:**
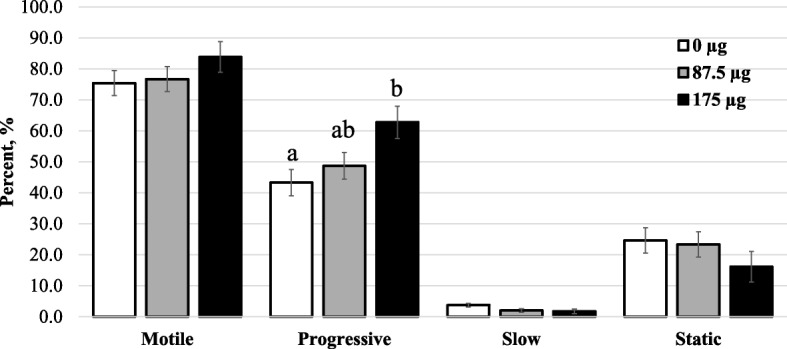
Motility of nanoselected spermatozoa after dose-dependent labeling/removal procedures. Dose-dependent effects of MNP concentrations were observed. Different letters indicate significant differences between column within the same analyzed parameter (*P* = 0.034). Data are mean ± sem of at least four independent replicates

**Fig. 5:**
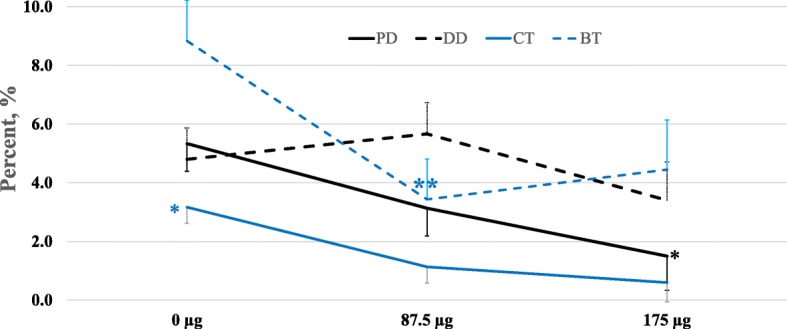
Morphology of nanoselected spermatozoa after dose-dependent labeling/removal procedures. Asteriks indicate significant differences between MNP concentrations, within the same parameters corresponding to proximal cytoplasmic droplet (PD), distal cytoplasmic droplet (DD), coiled tail (CT), and bent tail (BT). *P < 0.05 indicates difference from other groups and **P = 0.04 indicates difference from the control only. Data are mean ± SEM of at least four independent replicates

Results are summarized in Fig. 4 and Table 1. In this one-step nanoselection process, corresponding to the retrieval of non-apoptotic or acrosome intact, the presence of MNP conjugates was associated with dose-dependent increases in motile (*P* <  0.05) and forward progressive spermatozoa (*P* = 0.03), as well as VAP and VSL (*P* < 0.05) and VCL (*P* = 0.09). These effects contrasted with the decreased proportions of slow and static spermatozoa (Fig. 4 and Table 1). The proportions of sperm with morphological abnormalities (proximal droplets and coiled and bent tails) were dose-dependently decreased by the presence of MNP conjugates (Fig. 5; *P* < 0.05). Annexin V-MNP conjugates showed similar results and data are therefore not presented. The targeting of 1.6–2.0 × 10^9^ spermatozoa in 40 mL extender with 87.5 μg (~ 218,750 particles appeared as an optimal ratio that was used for the next experiments.

**Table 1:** Dose-dependent effect of MNP- lectin conjugates on sperm motility characteristics

Other motion characteristics	Nanoparticles (μg) per 40 mL of extended semen			*P*-value
	0 (*N* = 18)	87.5 (*N* = 19)	175 (*N* = 15)	
VAP, μm/s	63.9 ± 4.7^a^	77.6 ± 4.7^a^	88.8 ± 5.8^b^	0.04
VSL, μm/s	45.4 ± 2.3^a^	49.9 ± 2.3^a^	61.6 ± 2.8^b^	< 0.03
VCL, μm/s	128 ± 11.8	156.6 ± 11.8	173.5 ± 14.4	0.09
ALH, μm	5.4 ± 0.6	6.4 ± 0.6	6.6 ± 0.7	NS
BCF, Hz	38.9 ± 1.3	38.3 ± 1.3	36.6 ± 1.6	NS
STR, %	73.9 ± 3.3	68.4 ± 3.3	71.9 ± 4.0	NS
LIN, %	38.8 ± 2.7	36.3 ± 2.7	39.0 ± 3.3	NS

Table represents various sperm kinematic parameters, such as average path velocity (VAP), straight-line velocity (VSL), curve-linear velocity (VCL), head lateral amplitude (ALH), beat flagellum frequency (BCF), straightness (STR – VSL/VAP × 100), linearity (LIN – VSL/VCL × 100). Data are mean (±SEM) of N observations of at least 4 independent replicates using pooled semen doses. Different letters on the same line denote significant differences (*P* < 0.05)

#### Time-dependent incubation

**Fig. 6:**
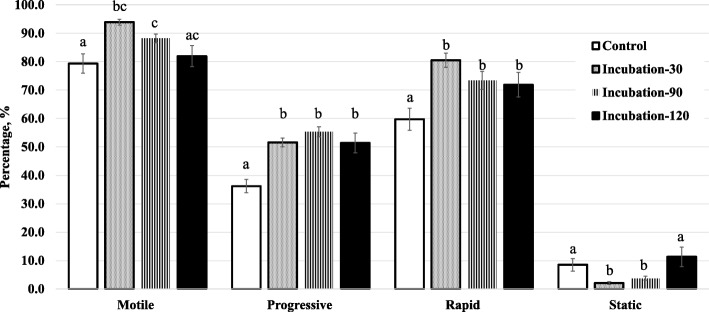
Motility of nanoselected spermatozoa after dose-dependent incubations. Different letters indicate significant differences between columns (incubation times) of each motility parameter (*P* < 0.001: total motility and progressive, *P* = 0.001: rapid and *P* = 0.02: static). Data are mean ± SEM of at least four independent replicates

Results are shown in Fig. 6 and Table 2. In this one-step nanoselection process, corresponding to the retrieval of non-apoptotic or acrosome intact, all incubation times showed overall benefits on sperm motility characteristics such as the proportions of motile, progressive, and rapid spermatozoa that were significantly increased in comparison to the control (Fig. 6; *P* ≤ 0.001 – Table 2; *P* = 0.02). Incubations of 90 min or lower generally increased the sperm velocity (VAP, VSL, and VSL) and kinematic (ALH, BCF, STR, and LIN) parameters (Table 2; *P* < 0.0001); however, no (Fig. 6) or moderate (Table 2) effects on various motility characteristics were observed after 120 min incubation. Incubations with Annexin V-MNP conjugates showed similar results and we choose not to present the data. Overall, the co-incubation of spermatozoa (1.6–2.0 × 10^6^ /40 mL) with either MNP conjugate (87.5 μg, ~ 218,750 particles) exhibited highest effectiveness after 30 min incubation. This co-incubation time was used for the next experiment.

**Table 2:** Time-dependent effect of MNP-lectin conjugates on sperm motility characteristics

Other motion	Incubation time, min				*P*-values
parameters	0 (N=18)	30 (N=19)	90 (N=15)	120 (N=16)	
VAP, μm/s	70 ± 2^a^	77.6 ± 2.1^b^	73.2 ± 3.1^c^	74.8 ± 1.7^bc^	< 0.0001
VSL, μm/s	35.4 ± 1.1^a^	40.5 ± 0.8^b^	45.1 ± 0.7^c^	42.7 ± 1.3^d^	< 0.0001
VCL, μm/s	154.5 ± 4.1^a^	164.7 ± 4.3^b^	150.3 ± 4.8^a^	160.4 ± 2.9^b^	0.001
ALH, μm	8.3 ± 0.1^a^	7.7 ± 0.1^b^	7.2 ± 0.3^c^	7.9 ± 0.1^b^	< 0.0001
BCF, Hz	38.1 ± 0.4^a^	36.2 ± 0.5^b^	37.9 ± 0.9^a^	34.1 ± 0.6^c^	< 0.0001
STR, %	51.1 ± 0.6^a^	54.2 ± 0.9^b^	63.1 ± 2.5^c^	58.1 ± 1.0^d^	< 0.0001
LIN, %	24.3 ± 0.5^a^	26.9 ± 0.5^b^	33.1 ± 2.2^c^	28.2 ± 0.6^b^	< 0.0001

The table represents various sperm kinematic parameters, such as average path velocity (VAP), straight-line velocity (VSL), curve-linear velocity (VCL), head lateral amplitude (ALH), beat flagellum frequency (BCF), straightness (STR – VSL/VAP × 100), linearity (LIN – VSL/VCL × 100). Data are mean (±sem) of N observations of at least 4 independent replicates using pooled semen doses. Different letters on the same line denote significant differences (*P*<0.05)

#### Two-step targeting

**Fig. 7:**
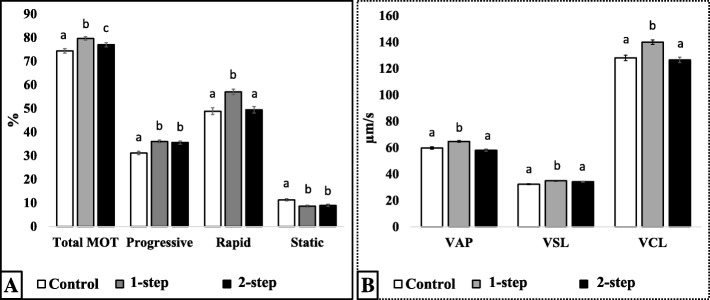
Motility (A) and velocity (B) characteristics of nanoselected spermatozoa following double or two-step removal. Total motile (total MOT), progressive (forward moving), rapid (fast), and static (dead/non-motile) spermatozoa with their corresponding average path (VAP), straight line (VSL), and curvelinear (VCL) velocities were assessed. Spermatozoa were standard non-selected (control) or successively nanoselected with Annexin-V (1-step) followed by Lectin (2-step) -MNP conjugates. Columns with different letters differ significantly (*P* < 0.05). Data are mean ± SEM of at least four independent replicates

In this process, the optimal conditions were used for sperm selection, consisting of 30 min co-incubation of extended semen (1.6–2.0 × 10^9^ spermatozoa/40 mL) with 87.5 μg of each MNP conjugate. Data presenting both 1-step (non-apoptotic) and 2-step (both non-apoptotic and acrosome intact) nanoselection process are summarized in Fig. 7 and Table 3 . In comparison to the control group, each nanoselection step (1-step or 2-step) significantly increased the proportions of total motile, forward progressive, and rapid spermatozoa, while significantly decreased the proportions of static spermatozoa (*P* < 0.05; Fig. 7a). The first step (1-step, removal of apoptotic) had stronger effects on sperm motility (total and rapid) and velocity parameters (VAP, VSL, and VCL; Fig. 7b) than the second step (2-step; *P* < 0.05). Directionality parameters were variably increased following each removal step (*P* < 0.05; Table 3), and the 2-step removal significantly decreased ALH while increasing the straightness (STR) and linearity (LIN) of spermatozoa when compared to control and 1-step counterparts.

**Table 3:** Additional movement characteristics of double nanoselected boar spermatozoa

	ALH, μm	BCF, Hz	STR, %	LIN, %
Control	7.95 ± 0.08^a^	38.14 ± 0.3^ab^	55.7 ± 0.6^a^	27.3 ± 0.4^a^
1-step removal^1^	8.06 ± 0.05^a^	37.6 ± 0.3^b^	55.8 ± 0.6^a^	27.3 ± 0.4^a^
2-step removal^2^	7.5 ± 0.1^b^	38.7 ± 0.3^a^	60.4 ± 0.8^b^	29.8 ± 0.7^b^

^1^Removal of apoptotic spermatozoa (annexin V-MNP conjugates = 1-step) was followed by those with ^2^acrosome reacted (lectin-MNP conjugates = 2-step). This consecutive removal process corresponds to the proposed double nanoselection with evaluation of the lateral head amplitude (ALH), beat cross frequency (BCF), straightness (VSL/VAP × 100), and linearity (VSL/VCL × 100). Data are mean (±sem) of 4 independent replicates; different superscripts (^a,b^) within the same column indicate significant differences (*P*<0.05)

#### Sperm retrieval

We used the SpermaCue (Minitube USA, Inc., Verona, USA) to evaluate the total sperm counts before and after the two-step targeting process. The results indicated that approximately 3% of spermatozoa were entrapped (~ 96–120 × 10^6^) during the two-step targeting, leaving 97% of free spermatozoa in each insemination dose (80 mL).

### Viability assessment of nanoselected spermatozoa

Analyses were performed on the 2-step nanoselected spermatozoa (Table 4). The proportions of spermatozoa stained for acrosome reaction (PNA-FITC: 92% ± 7.4% vs. 98% ± 7.4%), plasma membrane integrity (PI: 93% ± 8% vs. 94% ± 8%), mitochondrial potential (JC-1: 86% ± 13% vs. 88% ± 15%), and ROS accumulation (H_2_-DCFDA: 99.8% vs. 99.4%) were not significantly different between control and nanoselected spermatozoa, respectively (*P* > 0.05). However, relative fluorescence intensities (RFI) associated with ROS production and plasma membrane integrity in nanoselected spermatozoa were lower, but not significantly to the controls (864 ± 200 vs. 1069 ± 200 and 9 ± 26 vs. 12 ± 26, respectively; *P* > 0.05). Nanoselected spermatozoa showed non-significantly lower JC-1 fluorescence intensity than the control (898 ± 224 vs. 426 ± 200, *P* > 0.05).

**Table 4:** Viability assessment of nanoselected spermatozoa by Flow cytometry

	Control^1^	Nanoselected^2^
Reactive oxygen species	1,069 ± 200	864 ± 200
Plasma membrane integrity	11.9 ± 25.6	8.6 ± 25.6
Mitochondrial integrity	426 ± 200	898 ± 224

^1^Non selected (Control) vs. ^2^double-selected (annexin V and lectin; Nanoselected) spermatozoa. Data are relative fluorescence intensities (mean±sem) of spermatozoa stained with H_2_-DCFDA (ROS production), propidium iodide (plasma membrane integrity), or JC-1 (mitochondrial potential). Fluorescence intensity values are proportional to the extent of damages of 4 independent replicates (mean±sem). Data within the same line are not significantly different (*P*>0.05)

### Reproductive outcomes of nanoselected spermatozoa

Pregnancy and farrowing rates resulted for gilts inseminated with control [43% (3/7) and 100% (3/3)] and nanoselected [57% (4/7) and 100% (4/4)] spermatozoa were comparable (*P* > 0.05). Similarly, litter size and weight for gilts inseminated with control (11.3 ± 5.6 and 1.6 ± 0.04 kg, respectively) and nanoselected (12.7 ± 7.3 and 1.5 ± 0.04 kg, respectively) spermatozoa were comparable (*P* > 0.1), as were the proportion of live pigs weaned in litters of sows inseminated with control (89%; 40/45) and nanoselected (92%; 35/38) spermatozoa.

The glucose (56 ± 8 vs. 48 ± 8 mg/dL), hematocrit (22% ± 1% vs. 22% ± 2%), and IgG (342 ± 108 vs. 568 ± 112 ng/mL) measurements, all indicative of the neonatal pigs health performance, were not significant different between nanoselected- and control-born pigs. Likewise, no significant interactions were found between sperm treatment and offspring gender (*P* > 0.05).

All morphometric parameters showed similar measurements in both control- and nanoselected-born pigs (*P* > 0.05). All born pigs grew at normal and comparable pace, until weaning at 28 d of age (8.2 ± 0.2 kg vs. 7.7 ± 0.2 kg for control and nanoselected, respectively). However, the average head length was significantly shorter in nanoselected-born pigs vs. the controls, at the time of weaning (15.0 ± 0.2 cm vs. 16.2 ± 0.2 cm, *P* < 0.01), and there were no significant interactions between sperm treatment (control or nanoselected) and the offspring gender (*P* > 0.1).

## Discussion

Semen contain variable proportions of damaged spermatozoa that can be exacerbated during hot seasons, causing substantial productivity losses in commercial studs due to the rejection of poor semen [3, 34]. Numerous in vitro tests are being used to evaluate apoptosis and early acrosome reaction, two main causes of poor semen quality [11, 39, 40]. Obtained results are mainly informative [11, 21], and semen not meeting the minimum standards are still rejected. Here we showed that specifically designed magnetic nanoparticle conjugates could be used for smooth, effortless, and high-throughput targeting and removal of damaged spermatozoa from semen doses, without further affecting the functionality and fertility of nanoselected spermatozoa.

In this study, the synthesized iron oxide particles measured 7 to 14 nm in diameters, and corresponded to maghemite (γ-Fe_2_O_3_) and hematite (α-Fe_2_O_3_) as revealed by the HRTEM images showing respective interplanar distances of 2.54 Å and 2.98 Å [41, 42]. All forms of MNP (hematite or α-Fe_2_O_3_, maghemite or γ-Fe_2_O_3_, and magnetite or Fe_3_O_4_) have been used for in vitro targeting of spermatozoa, in a broad range of applications (i.e., sperm nanopurification, toxicity test, and sperm-mediated gene transfer), without affecting their functionality or structures (reviewed by [43]). In the current study, the conjugation of maghemite-made MNP with annexin V and hematite-made MNP with lectins (PNA/LCA) to respectively target apoptici and acrosome reacted spermatozoa [44–46] did not affect the MNP magnetism nor the functionality of both MNP conjugates. Various imaging technologies (MFM/AFM, TEM and hyperspectral) were used to confirm the presence of MNP conjugates in the sperm head, the expected localization of both defects. Both single- and multi-point attachments of MNP conjugates were observed on spermatozoa, and likely reflected the extent of damages. From this observation, it was speculated that deep damages may attract high number of MNP-conjugates, creating aggregates and necessary differential magnetism force for effective removal of damaged spermatozoa.

Previous studies have used the abovementioned MNP forms to harmessly remove apoptotic spermatozoa from boar and bull semen [29, 31, 47]. Fixed ratios of Lectin-MNP per boar (1 mg/33 × 10^6^) [29], and per bull (1 mg/10^6^) [31] spermatozoa were used, and resulting nanoselected spermatozoa led to full-term pregnancies and birth of healthy offspring. In contrast, the removal of both apoptotic and acrosome reacted spermatozoa was tested in the current study. We first conducted dose-dependent and time-dependent experiments to determine the optimum sperm-to-MNP conjugate combinations, the 2-step protocol. With the expectation to target approximately 20% of damaged spermatozoa per semen dose (80 mL containing 3–4 × 10^9^ spermatozoa), we added the equivalent volumes of 87.5 μg (87.5 μL) and 175 μg (175 μL) of each MNP conjugate into 40 mL of extended semen (1.5–2 × 10^9^ spermatozoa). Separate incubations (10–15 min) with 87.5 μg of each MNP conjugate had no effects on sperm motility characteristics, but significantly reduced the proportions of abnormal spermatozoa. Using 87.5 μg of each MNP conjugate, to avoid MNP toxicity [48, 49], all time-dependent incubations (30 to 120 min) were beneficial to sperm motility characteristics (vs. the control). The significantly decreased proportions of static spermatozoa after 30–90 min incubation indicated the ability of MNP conjugates to reduce the percentages of defective spermatozoa within the semen samples. This observation is of great interest as the accepted proportion of damage spermatozoa in boar studs varies from 15% to 30% [50, 51]. Altogether, the optimal condition for successful sperm nanoselection in the current study was summarized as 87.5 μg of MNP conjugate (per 40 mL of extended semen, 1.5-2.0 × 10^9^ spermatozoa), for 30–60 min incubation.

Investigating the efficiency of each MNP conjugate on sperm selection revealed a simultaneous elimination of apoptotic spermatozoa in 1-step, followed by acrosome-reacted spermatozoa in 2-step. This sequential process showed maximum sperm removal during the 1-step, indicating that removed spermatozoa may carry both apoptotic and acrosome damages. Both MNP conjugates (in 2-step) resulted in approximately 3% of sperm retrieval, corresponding to 96  – 120 × 10^6^/ insemination dose. This low removal rate may be due to the use of fresh semen harvested from high reproductive boars. Speculating that damaged spermatozoa constitute physical impediments within the semen dose, their successful removal increases the proportion of healthy and viable (nanoselected) spermatozoa, leading to high-quality semen doses available for artificial inseminations. These findings are consistent with the unique work in swine using a one-step nanoselection method with Lectin-MNP [29]. In support of the positive outcomes of the nanoselection process, sperm kinematic parameters such as VSL, STR, and LIN have shown positive correlations with fertilization rates [52], while being used as predictors of effective and smooth sperm displacement within the utero-oviduct tube [53, 54] and male fertility [55].

Altogether, the findings indicated the benefit of the proposed two-step nanoselection, showing complementary and synergy effects of the sequential removal. The 1-step removal (Annexin V-MNP) resulted in improved sperm motility (total, progressive, rapid, and static) and velocity (VAP, VSL, and VCL) parameters, while the 2-step (Lectin-MNP) was advantageous to ALH, BCF, STR, and LIN. Using similar Lectin-MNP, data of 1-step removal were comparable to a previous study with boars [29], which support the proposed sequential process to achieve optimum removal of moribund spermatozoa from semen doses.

Semen enrichment with high-quality nanoselected spermatozoa did not translate into higher viability than their control counterparts. The use of high-quality fresh spermatozoa exhibiting minimal damages may explain this inconsistency. Nanoselected spermatozoa maintained more stable plasma membrane, higher mitochondrial membrane potential, and lower ROS level. These features are vital for effective fertilization [56] and cryotolerance [57, 58]. A previous study has reported the positive effects of the SLC selection protocol on the freezability of boar spermatozoa and the decreased ROS production post-thaw [38]. These observations prompted us to field fertility trials, which did not reveal significant advantages of the nanoselection in fertility outcomes. The absence of plasmatic biochemical changes between piglets born from control or nanoselected spermatozoa allowed us to rule out any health or toxicity concerns.

## Conclusions

The proposed two-step or sequential nanoselection process allowed for 1) successful molecular-based targeting of apoptotic (Annexin V-MNP) and acrosome-reacted (Lectin-MNP) spermatozoa, 2) effortless, rapid, and high-throughput enrichment of semen doses with best spermatozoa, capable to induce normal pregnancy and post-natal development of piglets. The application of this process in studs is likely to decrease semen rejection rates, therefore increasing the potential of using boars with reduced semen quality.

## Abbreviations

Å: Angström (= 0.1 nm)
AFM/MFM: Atomic/Magnetic force microscopy
DLS: Dynamic light scattering
HRTEM: High resolution transmission electron microscope
MNP: Magnetic nanoparticles
MOT: Total motile sperm
PROG: Progressive motile sperm
RAP: Rapid motile spermatozoa
RFI: Relative fluorescence intensities
ROS: Reactive oxygen species
STAT: Static spermatozoa
STR: Straightness spermatozoa
TEM: Transmission electron microscope
VAP: Average path velocity
VCL: Curvelinear velocity
VSL: Straight-line velocity
